# Evaluation of fully automated motion corrected first pass myocardial perfusion MRI with semi quantitative perfusion parameter maps in patients with ischemic heart disease

**DOI:** 10.1186/1532-429X-14-S1-P249

**Published:** 2012-02-01

**Authors:** Aya Kino, Christopher Glielmi, Andrada R Popescu, Mauricio S Galizia, Jeremy Collins, Darshit Thakrar, Jacob Fluckiger, Hui Xue, Jens Guehring, Sven Zuehlsdorff, Daniel C Lee, James Carr

**Affiliations:** 1 Radiology, Northwestern University, Chicago, IL, USA; 2 Siemens Healthcare, Chicago, IL, USA; 3 Siemens Corporate Research USA, Princeton, NJ, USA; 4 Dept Medicine-Division of Cardiology, Northwestern University, Chicago, IL, USA

## Summary

The purpose of the study is to evaluate a fully automated motion corrected first pass myocardial perfusion (FPMP) MRI with semi quantitative perfusion parameter maps in patients with suspected ischemic heart disease.

## Background

Coronary heart disease is the leading cause of death and disability in the US. FPMP MRI is increasingly used to assess ischemic heart disease; however respiratory motion is one of the major problems for myocardial blood flow quantification. An algorithm for motion correction, surface coil correction, temporal denoising and robust pixel-wise parameter map generation model was previously desribed [Xue H et al MICCAI 2009).This work evaluates automated workflow in a clinical setting to diagnose ischemic heart disease comparing free breathing and motion correcteted images and corresponding pixel-wise parameter map.

## Methods

Stress and rest FPMP images were acquired using a 1.5T scanner (MAGNETOM Avanto, Siemens Healthcare) in 39 patients with suspected ischemic heart disease. Short axis slices were acquired during infusion of 0.075 mMol/kg of Gadolinium (Magnevist, Bayer HealthCare Pharmaceuticals, USA) and adenosine (Adenoscan, AstellasPharma, USA) infusion (0.14 mg/kg/min; duration: 4 min) was administrated to induce stress. Free breathing, motion-corrected images and corresponding perfusion maps were assessed by 2 radiologists independently using the AHA 16 model and evaluated using a four point Likert scale (poor to excellent) to evaluate image quality and confidence level in presence or absence of hypo-perfusion regions. Upslope index of both free breathing and motion corrected images during stress and rest were manually calculated in non-ischemic and ischemic areas and compared to the corresponding pixel-wise parameter map generated based on motion corrected images. FPMP MRI results were subsequently compared to coronary angiogram, stress echocardiography, or SPECT.

## Results

**Figure 1:**
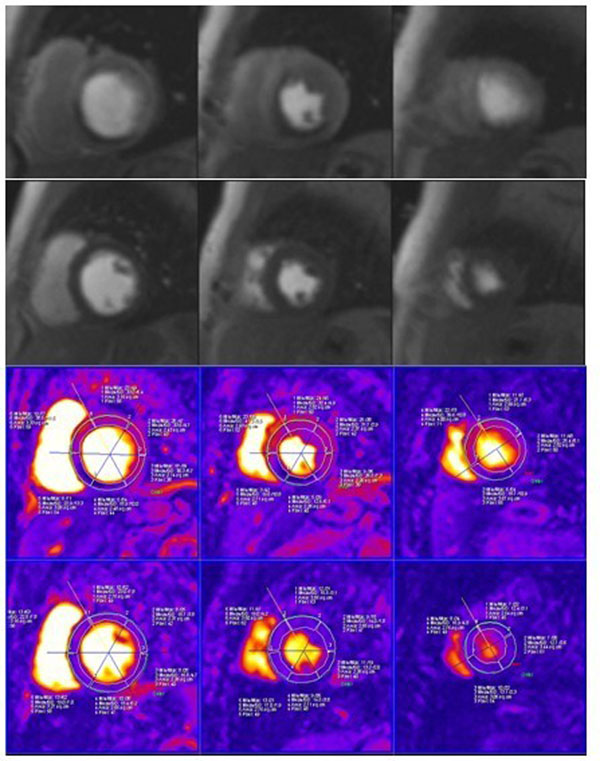
Perfusion images during the Stress (upper) and Rest (lower) and respective slope map from 57 years old woman with previous history of coronary artery disease post CABG with angina. Perfusion defects are seen at the basal mid and apical septal inferior and inferior regions corresponding to a RCA and LAD stenosis and map slopes values are not significant different during stress and rest images at the corresponding segments. Her coronary angiography examination showed 100 % stenosis at LAD with LIMA graft patent, 70% in stent stenosis and 80 %stenosis at RCA.

Perfusion defects were detected in 25 patients (representative patient shown in Fig. 1). Mean image quality score for motion corrected images (3.48 ± 0.50) and confidence level (3.31 ± 0.41) were significantly higher (p<0.001) than free breathing images (mean image score of 2.51 ± 0.63 and confidence level of 2.83 ± 0.58). Upslope index of non ischemic and ischemic areas and semi quantitative perfusion parameter maps values were comparable (P> 0.05). Sensitivity and specificity for each technique, as well as the inclusion of perfusion parameter maps, are shown in Table 1. Although inline processed results showed higher sensitivity and specifity than standard images, differences were not significant (p< 0.05).

**Table 1:** Sensitivity and Specificity values from both readers by technique per subject.

Reader 1	FB	MC	MC + map	Reader 2	FB	MC	MC + map
Sensitivity	0.85	0.89	0.89	Sensitivity	0.89	0.91	0.91
Specificity	0.82	0.85	0.85	Specificity	0.87	0.90	0.90

Sensitivity and Specificity by reader and by technique. There is a increase in sensitivity and specificity during MC images and MC + map images.

## Conclusions

A fully automated motion corrected first pass myocardial perfusion (FPMP) MRI with semi quantitative perfusion parameter maps showed comparable accuracy for detection of significant coronary artery disease in patients with ischemic disease.

## Funding

Astellas Pharma Global Development, Inc.

